# Modified Hybrid Procedure in Hypoplastic Left Heart Syndrome: Initial Experience of a Center in Northeastern Brazil

**DOI:** 10.21470/1678-9741-2017-0058

**Published:** 2017

**Authors:** Renato Max Faria, Juliana Torres Pacheco, Itamar Ribeiro de Oliveira, José Madson Vidal, Anilton Bezerra Rodrigues Junior, Ana Luiza Lafeta Costa, Vinicius José da Silva Nina, Marcelo Matos Cascudo

**Affiliations:** 1 Hospital Wilson Rosado, Mossoró, RN, Brazil.; 2 Casa de Saúde São Lucas, Natal, RN, Brazil.; 3 Departamento de Cirurgia Cardiovascular do Instituto do Coração de Natal (Incor/Natal), Natal, RN, Brazil.; 4 Universidade Federal do Maranhão (UFMA), São Luís, MA, Brazil.

**Keywords:** Hypoplastic Left Heart Syndrome, Congenital Abnormalities, Bandages, Angioplasty, Contrast Media

## Abstract

**Introduction:**

Although it only corresponds to 2.5% of congenital heart defects, hypoplastic left heart syndrome (HLHS) is responsible for more than 25% of cardiac deaths in the first week of life. Palliative surgery performed after the second week of life is considered an important risk factor in the treatment of HLHS.

**Objective:**

The aim of this study is to describe the initial experience of a medical center in Northeastern Brazil with a modified off-pump hybrid approach for palliation of HLHS.

**Methods:**

From November 2012 through November 2015, the medical records of 8 patients with HLHS undergoing hybrid procedure were retrospectively evaluated in a tertiary private hospital in Northeastern Brazil. The modified off-pump hybrid palliation consisted of stenting of the ductus arteriosus guided by fluoroscopy without contrast and banding of the main pulmonary artery branches. Demographic and clinical variables were recorded for descriptive analysis.

**Results:**

Eight patients were included in this study, of whom 37.5% were female. The median age and weight at the time of the procedure was 2 days (p25% and p75% = 2 and 4.5 days, respectively) and 3150 g (p25% and p75% = 3077.5 g and 3400 g, respectively), respectively. The median length in intensive care unit stay was 6 days (p25% and p75% = 3.5% and 8 days, respectively). There were no in-hospital deaths. Four patients have undergone to the second stage of the surgical treatment of HLHS.

**Conclusion:**

In this series, the initial experience with the modified off-pump hybrid procedure showed to be safe, allowing a low early mortality rate among children presenting HLHS.

**Table:** 

Abbreviations, acronyms & symbols	
ASD	= Atrial septal defects
DATASUS	= Departamento de Informática do Sistema Único de Saúde (Department of Informatics of the Brazilian Unified Health System)
HLHS	= Hypoplastic left heart syndrome
ICU	= Intensive care unit
PGL2	= Prostaglandin
PTFE	= Polytetrafluoroethylene

## INTRODUCTION

Although it only corresponds to 2.5% of congenital heart defects, hypoplastic left heart syndrome (HLHS) is responsible for more than 25% of deaths from congenital heart disease in the first week of life^[1]^. Ninety-five percent of patients with this condition, if not submitted to palliative surgery, evolve to death in the first month of life^[2]^.

The management of HLHS continues today as one of the major challenges in pediatric heart surgery. The experience gained over the past 20 years, including an improvement in the medical and surgical management of this condition, has elected the surgical staging as preference in most centers worldwide. However, the Norwood operation for HLHS has a significantly higher mortality than other surgeries performed in the neonatal period^[3]^.

Given the high initial mortality in Norwood procedure, some centers in the 90s developed an alternative option, known as "hybrid procedure" (by the combination of a surgical procedure and an interventional catheterization), consisting of a stent implant in the ductus arteriosus to keep it open, associated with regulation of pulmonary blood flow through the banding of the pulmonary artery branches, and septostomy of the interatrial septum. The "hybrid" route for HLHS has been developed in centers with high mortality in the Norwood operation, due to its lower eventual mortality in the initial stage and its potential advantage in avoiding circulatory arrest and deep hypothermia inherent to the Norwood operation^[4,5]^.

Thus, the hybrid procedure for handling HLHS was developed as an alternative to the Norwood procedure, in the neonatal period, providing a less invasive initial palliative treatment and, in most cases, with lower costs, becoming a great therapeutic option in an attempt to reduce mortality associated with the Norwood procedure^[6]^.

Countless variables have been associated with the increased surgical mortality rate. Palliative surgery performed after the second week of life is considered an important risk factor in the treatment of HLHS^[7]^.

The prenatal diagnosis also helps to improve survival after the first stage of the palliative surgery when compared to postnatal diagnosis, since it allows a better child labor arrangement, which shall be conducted in an appropriate referral facility, besides the early infusion of prostaglandin (PGL2), to help maintain a systemic cardiac output through the ductus arteriosus and, consequently, maintain the newborn clinically stable for carrying out the first stage of the heart surgery^[8]^.

The Brazilian Northeast region is needy for specialized care for the treatment of congenital heart diseases. According to DATASUS (Department of Informatics of the Brazilian Unified Health System), there is an annual deficit of 75.1% on surgical procedures for children with congenital heart disease in the region. Associated with this matter, the transfer of these children to hospitals in the Brazilian Southeast region depends on a national referral policy, which requires an extended holding time, resulting in the worsening of prognosis of the treatment or even in the child's death^[9]^.

Based on its lower cost, no need for cardiopulmonary bypass and the little experience of our group with the Norwood surgical technique, we decided to start a modified hybrid treatment for HLHS at the end of 2012.

The aim of this study is to describe the initial experience of a center in the Brazilian Northeast region with a modified offpump hybrid palliation for HLHS, which consists in a combined intraoperative stenting of the ductus arteriosus without contrast and banding of the main pulmonary artery branches.

## METHODS

### Study Design

From November 2012 through November 2015, the medical records of all patients diagnosed with HLHS who underwent the hybrid procedure were retrospectively evaluated. This study was carried out in a tertiary private hospital of the Northeast Brazilian region, which provides services to the Unified Health System in a complementary way.

### Variables

Demographic and clinical variables were recorded for descriptive analysis, which included: age, sex, weight, intensive care unit (ICU) length of stay, mortality and progression to the second stage of the surgical treatment (Norwood-Glenn procedure). Early mortality was defined as death occurring within 30 days of surgery or before hospital discharge.

### Statistics

A descriptive analysis of data was performed. Qualitative variables were expressed by percentage and quantitative variables by median and percentile (p25% and p75%).

### Ethics

Written informed consent was obtained from the parents before all the hybrid procedures.

In accordance with the Resolution 466/12 of the MOH of Brazil, this case series study was approved by the Ethics Review Board under protocol number CAAE: 58105616.1.0000.5084 and Consolidated Report No. 1.665.194.

### Operative Technique

The modified hybrid procedure is usually performed in the catheterization laboratory under general anesthesia. The patient is monitorized with a 5-lead electrocardiogram, an arterial line inserted in the left radial artery, a 4 French venous central line in the right internal jugular vein, an esophageal thermometer probe and also a capnometer and a peripheral oxymeter for measurement of CO2 and oxygen saturation levels, respectively.

**Fig. 1:**
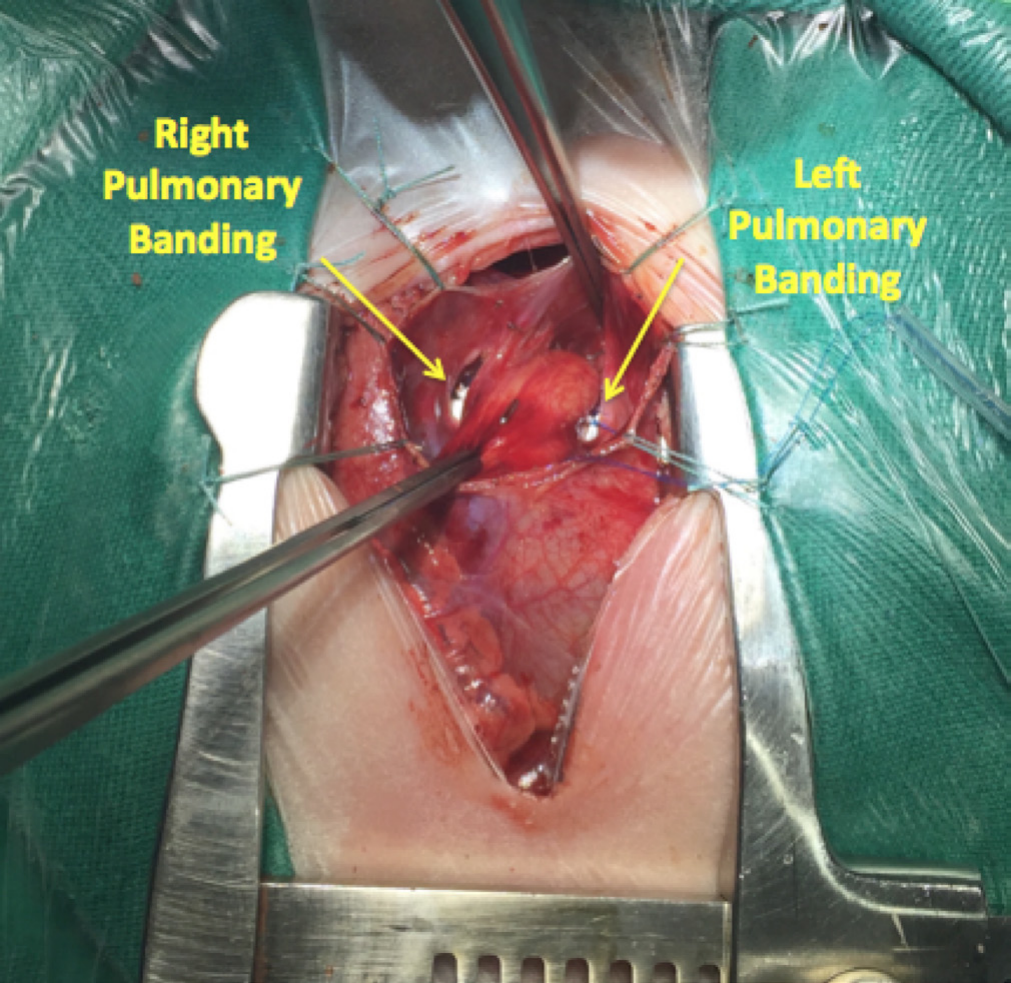
Final aspect of the pulmonary banding.

The approach is through a median thoracotomy. The procedure begins with the banding of the pulmonary artery branches using a 3.5 mm polytetrafluoroethylene (PTFE) graft, which we cut into a rectangular small piece of 3 x 22 mm. After wrapping the pulmonary artery branches, the banding is secured with a 6-0 Prolene^®^ suture (Ethicon, São Paulo, Brazil) in order to reduce 10 points from baseline oxygen saturation and consequently increase by 10 mmHg the baseline median arterial pressure. At the end, we cut the excess of PTFE. The final aspect of the banding is shown on Figure 1.

**Fig. 2:**
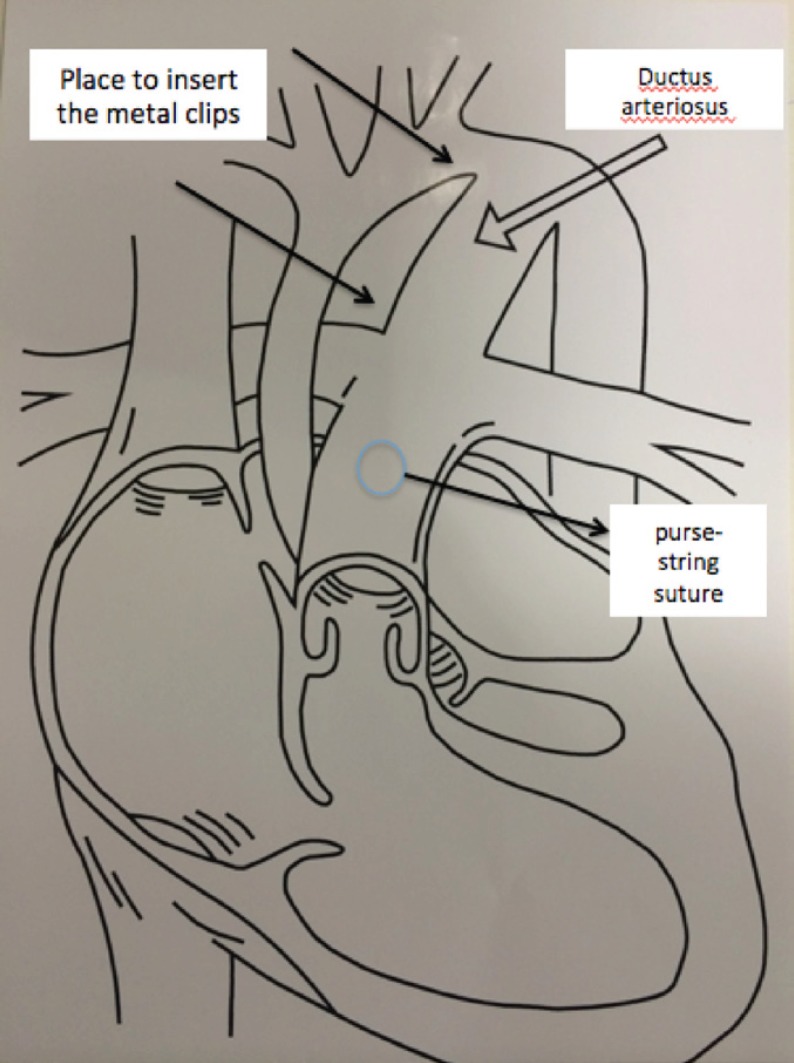
Placement of the metal clips and a circular 6-0 Prolene^®^ (Ethicon, Sao Paulo, Brazil) purse-string suture placed in the pulmonary trunk.

The measurement of the length and diameter of the proximal and distal end of the ductus arteriosus is done preoperatively using a transthoracic echocardiogram Vivid S5 (GE^®^, Fairfield, USA) with a 2.7-8 MHz pediatric transducer. These measurements facilitate the choice of the stent size. The adventitia of the origin and of the distal end of the ductus arteriosus is marked with metal clips to guide the stent deployment (Figure 2). After that, a circular 6-0 Prolene^®^ (Ethicon, São Paulo, Brazil) purse-string suture is placed in the pulmonary trunk, where the introducer is inserted, approximately 0.5 to 1 cm below the proximal end of the ductus arteriosus allowing the stent's release in the correct position (Figure 2).

**Fig. 3:**
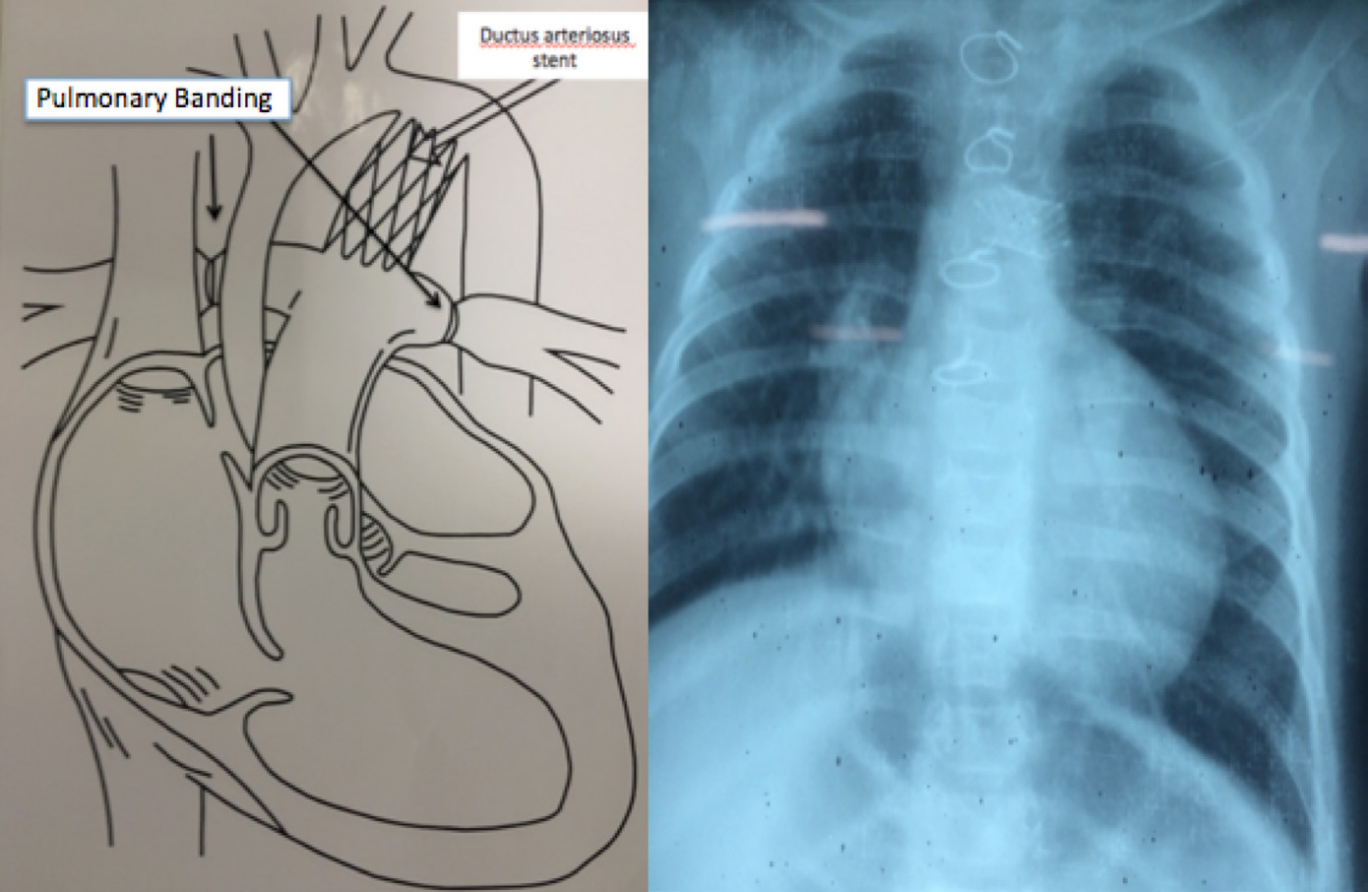
Final surgical and radiographic aspect of the hybrid procedure.

Under radioscopic view using Integris Allura 12C (Philips^®^, Amsterdam, Holland), the stent is deployed through the previously positioned introducer, having the adventitial clips as reference markers. Thus, this modification of the technique precludes the use of contrast and the need for peripheral arterial punctures and cardiopulmonary bypass. Figure 3 shows the final surgical and radiographic aspects of the modified hybrid procedure.

In all 8 cases, as the atrial septal defects (ASD) were not considered restrictive on the preoperative echocardiographic assessment, a balloon atrial septostomy was not performed. Serial echocardiograms were carried out at 30 days after discharge and every three months thereafter.

## RESULTS

Eight patients were included in this study, of whom 37.5% were female. The median age and weight at the time of the procedure was 2 days (ranging from 2 to 10 days) and 3150 grams (ranging from 2945 to 3600 grams), respectively. The median length of ICU stay was 6 days (ranging from 2 to 24 days).

There were no in-hospital deaths in this series. Two (25%) deaths occurred after hospital discharge, one of which was consequent to pneumonia two months after the procedure. The other case occurred three months after completion of the first stage of palliation, secondary to decompensated cardiogenic shock due to a restrictive atrial septal defect.

The mean follow-up was 138,6 days (60-284 days). Four (50%) children have successfully undergone to the second stage of the treatment of HLHS (Norwood-Glenn procedure), while two were still waiting to have this procedure done at the completion of this study, since they were only three months old at that time. All demographic and clinical data are summarized on Table 1.

**Table 1:** Epidemiological characteristics of patients presenting HLHS.

Patient	Date of birth	Age at procedure (days)	Weight	Gender	ICU stay (days)	2^nd^ stage
1	11/14/2012	2	3100 g	M	8	08/25/2013
2	06/20/2014	2	2945 g	F	5	Death (respiratory infection – 2 months)
3	05/20/2014	3	3055 g	F	4	11/11/2014
4	08/25/2014	2	3200 g	M	24	04/01/2015
5	03/08/2015	1	3100 g	M	8	Death (restrictive ASD – 3 months)
6	06/16/2015	2	3200 g	M	7	11/17/2015
7	08/08/2015	6	3600 g	M	3	Waiting
8	08/07/2015	10	3600 g	F	2	Waiting

## DISCUSSION

Even today, HLHS remains one of the most challenging pathologies among congenital heart diseases. Despite of the recent data reflecting a continuous improvement of the outcomes in the world's best centers of pediatric cardiology, the mortality rate is still high, especially in less experienced institutions^[10]^.

The advantage of the modified technique presented in this case series in comparison to the standard hybrid procedure^[4]^ resides on the fact that the insertion of the stent is conducted under fluoroscopic view using the metallic clips placed on the pulmonary artery wall as radiographic markers for its proper deployment, which precludes completely the use of any ionic or non-ionic contrast media.

The main difference is the stenting technique. On the original procedure, the stent is placed under angiographic control using radiologic contrast: "through a sheath in the main pulmonary artery, using angiographic control, an appropriate sized stent is placed"^[4]^; while in the current study, this technique was modified by deploying the stent under radioscopic view having adventitial metal clips as reference markers. Thus, this modification precludes the use of contrast as proposed on the original technique.

We have tried to perform the hybrid procedure as early as possible (median of two days of life). However, two of our patients who underwent the procedure at 6 and 10 days of life. Although some studies^[6]^ present an average age of about 7 days of life, in our experience this delay reflected the difficulty to obtain early diagnosis of the condition, as well as the failure to refer the pregnant women to deliver their children in one of the pediatric cardiac surgery specialized facilities of our region.

Previous study analyzing mortality, after the hybrid procedure, have shown a survival rate of 80% to 97%^[8]^. Our results have also shown favorable outcomes related to early mortality, since there were no deaths among the 8 patients analyzed. This fact has driven us to continue treating this complex heart disease in a mid-sized facility in the Northeast region of Brazil.

However, during clinical follow-up, there was one death due to pneumonia after hospital discharge. Another death occurred three months after completion of the first stage, secondary to decompensated cardiogenic shock due to a restrictive atrial septal defect, reflecting the difficulty of maintaining clinical follow-up continuity of our patients because of a lack of specialized professionals in the primary care network at our state. To minimize this matter, we have set up a specialized team for the treatment of complex pathologies^[12]^, since that there has been an increased number of cases referred to our care.

According to Honjo et al.^[11]^. the one-year survival is equivalent among children undergoing the Norwood and hybrid procedure (69.2% *vs.* 73.7%, respectively, *P*=0.83), however we have not found reports indicating if the length of hospital stay after these two approaches are also equivalent. In our study, we present a median length of stay in the ICU of 6 days.

Among the patients studied, 50% already performed the second surgical stage, and 2 patients were not at the appropriate age and weight to perform this procedure. Because of the deaths occurred, we have tried to keep the continuous monitoring, monthly, which was only possible after assembling a specialized multidisciplinary team, as suggested by Anuradha et al.^[12]^, to complement the assistance given to people in the public health care network.

The hybrid procedure has become the procedure of choice in many care facilities around the world because of neither requiring cardiopulmonary bypass nor deep hypothermic circulatory arrest; it can be performed in a shorter surgical time with lower risk of neurological damage, when compared to the Norwood surgery^[13]^.

We believe that the technical variant developed by our team is feasible and reproducible and gathers advantage to the original technique^[4]^ by avoiding the use of intravenous contrast for the stent implantation and by reducing the risks of complications with the administration of such compounds in low-weight patients.

The preliminary measurement of the ductus arteriosus by the echocardiogram and the use of metallic clips at its both ends help the stent deployment, avoiding the use of intravenous contrast. This advantage translates into a probable reduction of clinical complications; however, it will only be possible to confirm statistically this finding with a greater number of treated patients in our facility. Thus, the retrospective design and the small sample are the limitations of the current study.

Despite all technological advances, the treatment of HLHS still presents a challenge to physicians, especially in a region where 75% of children are not surgically treated^[9]^. Throughout the analysis of our results, we could glimpse a hope on the care for the complex heart diseases in northeastern Brazil.

## CONCLUSION

In this series, the initial experience with the modified offpump hybrid procedure showed to be safe, allowing a low early mortality rate among children presenting HLHS. However, advances are still needed to decrease the interstage mortality, as well as a larger number of cases to statistically prove the benefits of this technique.

Therefore, more efforts should be made to improve the current outcomes of this type of palliation and, ultimately, the prognosis of this subset of patients. Currently, after three years of experience, we have developed a less invasive technique which precludes the use of contrast media, which we hope will positively reflect on the long-term outcomes, as well as an increased interest of colleagues from other medical centers to adopt our approach for the treatment of this complex pathology.

**Table:** 

Authors' roles & responsibilities	
RMF	Analysis, or interpretation of data for the work; conception and design study; manuscript redaction or critical review of its content; final approval of the version to be published
JTP	Analysis, or interpretation of data for the work; final approval of the version to be published
IRO	Realization of operations and/or trials; final approval of the version to be published
JMV	Realization of operations and/or trials; final approval of the version to be published
ABRJ	Realization of operations and/or trials; final approval of the version to be published
ALLC	Analysis, or interpretation of data for the work; final approval of the version to be published
VJSN	Final approval of the version to be published
MMC	Realization of operations and/or trials; final approval of the version to be published
